# Epidemiology of suicide: recent developments

**DOI:** 10.1017/S2045796019000672

**Published:** 2019-11-07

**Authors:** Ross J. Baldessarini

**Affiliations:** Mailman Research Center, McLean Hospital, 115 Mill Street, Belmont, MA 02478-9106, USA

**Keywords:** Diagnosis, location, prevention, psychiatric illness, risk factors, suicide rates

## Abstract

Suicide and suicide attempts have become more prevalent in recent years, with notable increases in the US in all age groups and geographic locations. Risk of suicide is particularly high among patients diagnosed with bipolar disorder or severe depression, especially when associated with mixed features or agitation, or with co-occurring substance abuse. Factors contributing to such risk include relative social and geographic isolation and low access to sources of support or clinical care. In addition, unemployment, poverty, demoralisation and opioid abuse have been identified as important risk factors. Notably, overall longevity rates in the US, though rising for many decades, have recently been declining, in part owing to suicide and substance overdoses. A particular circumstance associated with strikingly high rates of suicides and attempts is the days and weeks following discharge from psychiatric hospitalisation. Although the incidence of such events is low, there is a need for more secure aftercare planning and implementation. Research on therapeutics aimed at reducing suicidal risk and all-cause mortality among psychiatric patients remains severely under-developed.

Excess mortality is a critical, under-appreciated outcome of major mental illnesses. Suicide is strongly associated with mood disorders, especially bipolar disorders and severe or melancholic major depression, and is particularly likely among adolescents and young adults, and with co-occurring substance abuse (Simon and Hales, 2012; Plemmons *et al*., 2018). In addition, mortality is increased by apparent accidents, complications of substance abuse and of co-occurring medical disorders, especially those sensitive to stress, including cardiovascular and pulmonary conditions (Ösby *et al*., 2001; Baldessarini *et al*., 2019*b*).

National rates of suicide have risen steadily in the US, from approximately 10.5/100 000 person-exposure-years (per 100k PEY) in 2003 to nearly 13.4/100k PEY in 2016, overall, and rates of fatal drug overdoses in the country increased by more than 4.4-fold from 1999 to 2015 (CDC 2018; Hedegaard *et al*., 2018). There have been wide geographic differences in suicide rates, from below 10/100k PEY in many coastal and urban regions, to over 36/100k PEY, in Alaska and parts of the inter-mountain western US (Hedegaard *et al*., 2018; Rossen *et al*., 2018). Nevertheless, from 2005 to 2015, US suicide rates rose consistently across rural, small-town, suburban and urban regions by 1.7–2.7-fold (Rossen *et al*., 2018). As in earlier years, relative suicidal risks within subgroups defined by sex or ethnicity have remained stable. They have been several-fold higher among men than women, and by ethnicity rank: Native American > Caucasian > Hispanic > African-American (CDC 2018). Factors contributing to these ominous trends may include demoralising effects of under-employment and poverty, as well as marked increases in opioid abuse (Hedegaard *et al*., 2018). These increases have also been offered as a plausible contribution to unprecedented, recently declining overall longevity in the US (Redfield, 2018). Of note, there is a striking disparity between sharply rising suicide rates in the US *v*. declining numbers of psychiatric beds in recent decades (Bastiampillai *et al*., 2016).

Clinical factors are important contributors to suicidal risk, and indeed most cases of suicide meet diagnostic criteria for a psychiatric illness (Simon and Hales, 2012). In our recent review of more than 6000 psychiatric patients who were well-evaluated over time, risks of suicide were highest among persons diagnosed with a DSM-5 bipolar disorder, with somewhat higher rates with type I than type II bipolar disorder, and greatest risks among those with mixed manic-depressive features such as agitated depression or dysphoric mania (RJ Baldessarini and L Tondo, unpublished observations, 2019). Intermediate rates were found with primary substance abuse disorders and chronic psychotic disorders, and the lowest rates with anxiety disorders (RJ Baldessarini and L Tondo, unpublished observations, 2019). The suicide rate in bipolar I disorder patients averaged 224/100k PEY approximately 15-times higher than general population rates which average about 15/100k/year. Moreover, suicidal acts were considered violent in 40% of bipolar I disorder patients, whereas violent acts were much less prevalent with other diagnoses and among women.

A noteworthy aspect of suicidal behaviour associated with major mental disorders is that the proportion of fatal outcomes of suicide attempts is much higher than that in the general population. We have proposed that the ratio of the rates of attempts/suicides (A/S) can serve as an *index of lethality* (Baldessarini *et al*., 2019a). This ratio has been over 30 in the general population (Kessler *et al*., 2005), and as low as 4.3 among bipolar I disorder patients (RJ Baldessarini and L Tondo, unpublished observations, 2019), indicating greatly increased lethality of intent or method with some psychiatric disorders. In the US, suicide by gunshot has been the leading method among men, and gunshot or self-poisoning among women (CDC 2018).

We also evaluated risk factors associated with suicidal behaviour among patients with specific major mood disorders diagnosed by modern DSM-5 criteria (Baldessarini *et al*., 2019*a*). Such factors can help to guide the need for clinical vigilance generally, although confident prediction of who will become suicidal and when remains elusive (Simon and Hales, 2012). We found the following factors to be especially highly statistically associated with suicidal behaviour in patients with bipolar or major depressive disorder: diagnosis of bipolar disorder > treatment with a mood-stabiliser or antipsychotic medicine > drug or alcohol abuse > separated or divorced > family history of suicide > mixed-manic-depressive features. Additional risk factors reflect relative social isolation and the lack of ready access to sources of support or clinical care (Tondo *et al*., 2006). A particularly ominous finding among bipolar disorder patients was that half of lifetime risk of suicidal behaviour occurred within the first 2–3 years of clinically manifest illness, whereas diagnosis and appropriate treatment of the disorder is typically delayed by 8–10 years from initial manifestations, and even longer following juvenile onset (Post *et al*., 2010; Tondo and Baldessarini, 2015).

An especially dangerous time for suicidal risk is immediately following discharge from psychiatric hospitalisation (Chung *et al*., 2017; Forte *et al*., 2019). We found an overall observed pooled rate of completed suicide within 12 months of hospital discharge of 241/100k PEY [CI: 238–243] in 41 studies involving a variety of psychiatric illnesses, and for attempts, 722/100k PEY [698–746] in 13 studies, indicating very high lethality (A/S  = 3.00) (Forte *et al*., 2019). This suicide rate is approximately 16-times higher than in the general population. In six studies (with 64 848 subjects) reporting on both suicides and attempts, the ratio (A/S) of annualised rates for attempts/completed suicides was 8.79 [6.63–12.0] – again, far lower than in the general population. Among all 48 studies, the cumulative distribution of suicidal events included 26.4% [25.9–26.9] within the initial month, 40.8% [40.2–41.4] by 3 months and 73.2% [72.7–73.7] within 12 months of discharge. The leading method of suicide attempts was by hanging or asphyxiation, rather than by gunshot or overdose as in the general population (Forte *et al*., 2019).

These findings indicate high risks of suicides and attempts soon after hospital discharge, with one-third of the one-year risk occurring within the initial two weeks. They strongly encourage more secure aftercare planning and immediate implementation following hospitalisation, particularly among patients with a severe mood, psychotic or substance abuse disorder, and those whose hospitalisation was indicated by emerging suicidal risk. A challenge to this recommendation of high vigilance and vigorous protection is that suicidal events, though much more prevalent following hospital discharge compared to other times in the course of mental illnesses, are still infrequent. We estimated that over 400 discharges would occur in order to encounter a single suicidal act within four weeks of hospital discharge (Forte *et al*., 2019).

The findings briefly reviewed here, arising largely from our recent research, indicate that suicide rates are increasing markedly, at least in the US. We are now able to state with greater confidence the association of suicidal behaviour with particular types of psychiatric illness based on current diagnostic criteria, to clarify risk factors for particular mood disorders, and to identify conditions associated with particularly high risk.

Proposed explanations for the observed recent, adverse, epidemiological trends summarised here remain impressionistic and incomplete. Moreover, means of effectively decreasing suicidal risk and of all-purpose mortality associated with major psychiatric illnesses remain unsatisfactory. Of particular concern, studies of therapeutic interventions specifically aimed at reducing suicidal risk are rare and extremely challenging to carry out in an ethical and scientifically credible manner. Indeed, only clozapine for schizophrenia has been recognised by the US FDA as having significant potential to reduce suicidal risk (Meltzer *et al*., 2003), and there is evidence to support such an effect of lithium treatment of severe mood disorders (Baldessarini *et al*., 2006; Guzzetta *et al*., 2007; Tondo and Baldessarini, 2015). In general, improved treatment of psychiatric disorders appears to reduce overall mortality, although much more research is required to improve overall mortality risks and for suicide in particular (Baldessarini, 2013).
